# Aging in an Increasingly Technology-Based Society: Past, Present, and Future

**DOI:** 10.1093/geroni/igaa057.2085

**Published:** 2020-12-16

**Authors:** Walter Boot

**Affiliations:** Florida State University, Tallahassee, Florida, United States

## Abstract

While it took over 70 years for the telephone to reach 50% of U.S. households, it took only 14 years for the cellphone, and 6 years for the MP3 player. Aging takes place in the context of a rapidly evolving technological landscape. What are the implications of such radical and rapid changes for how we age? And how can existing and emerging technologies help support aging adults’ health, wellbeing, social connectivity, and cognition? This talk will explore these issues, starting with the “digital divide” between younger and older adults, the reasons for this divide, and interventions to close the gap. The potential of emerging technologies to support older adults will be summarized, as well as potential pitfalls in the design and implementation of these technologies. The talk will conclude with speculation on the future of the digital divide and whether it can ever be entirely eliminated.

